# CircR2Disease: a manually curated database for experimentally supported circular RNAs associated with various diseases

**DOI:** 10.1093/database/bay044

**Published:** 2018-05-04

**Authors:** Chunyan Fan, Xiujuan Lei, Zengqiang Fang, Qinghua Jiang, Fang-Xiang Wu

**Affiliations:** 1 School of Computer Science, Shaanxi Normal University, Xi’an 710119, China; 2 Center for Bioinformatics, School of Computer Science and Technology, Harbin Institute of Technology, Harbin, Heilongjiang 150001, China; 3 Department of Mechanical Engineering and Division of Biomedical Engineering, University of Saskatchewan, Saskatoon, SK S7N 5A9, Canada

## Abstract

CircR2Disease is a manually curated database, which provides a comprehensive resource for circRNA deregulation in various diseases. Increasing evidences have shown that circRNAs play critical roles in transcriptional, post-transcriptional and translational regulation. Therefore, the aberrant expression of circRNAs has been associated with a group of diseases. It is significant to develop a high-quality database to deposit the deregulated circRNAs in diseases. The current version of CircR2Disease contains 725 associations between 661 circRNAs and 100 diseases by reviewing existing literatures. Each entry in the CircR2Disease contains detailed information for the circRNA–disease relationship, including circRNA name, coordinates and gene symbol, disease name, expression patterns of circRNA, experimental techniques, a brief description of the circRNA–disease relationship, year of publication and the PubMed ID. CircR2Disease provides a user-friendly interface to browse, search and download as well as to submit novel disease-related circRNAs. CircR2Disease could be very beneficial for researches to investigate the mechanism of disease-related circRNAs and explore the appropriate algorithms for predicting novel associations.

**Database URL**: http://bioinfo.snnu.edu.cn/CircR2Disease/

## Introduction

Circular RNAs (circRNAs) are a class of recently re-discovered endogenous non-coding RNAs (ncRNAs) and have been found in various tissues and cell lines across most lives including archaea, plants and animals (1–4). CircRNAs are mostly generated from back-splicing events, a process in which the downstream 5′ splice site and the 3′ splice site are covalently linked to form covalently closed loops (5, 6). Normally, circRNAs regulate gene expression at transcriptional or post-transcriptional levels by titrating microRNAs (miRNAs), regulating transcription and interfering with splicing (2, 7, 8). In recent years, with the applications of microarray, RNA-seq and other techniques, the deregulated circRNAs are widely detected in a broad spectrum of diseases, including gliomas (9, 10), esophageal cancer (11), hepatoma carcinoma (12) and so on. In addition, circRNAs have the characteristics of universality, tissue/cell specific specificity, conservatism, stability (13–16), as well as easy to detect in human blood (17) or saliva (18). Thus, circRNAs are becoming the ideal class of molecular biomarkers for disease diagnose and treatment.

Recent studies have constructed several databases for circRNAs. For example, CircBase merged and unified several circRNAs datasets into a standardized database, including circRNA IDs, genomic coordinates and best transcripts and so on. (19). The CircNet provided novel circRNAs, expression profiles, circRNA isoforms and circRNA-miRNA-mRNA regulatory networks (20). The Tissue-Specific CircRNA Database (TSCD) identified the tissue-specific circRNAs and characterized the features and functions of circRNAs (13). CircInteractome allowed researchers to search the potential interactions of circRNAs with RNA-binding proteins (RBPs) and miRNAs, as well as designed specific circRNA divergent primers and circRNA-directed siRNAs (21). The starBase systematically identified the RNA–RNA and protein–RNA interaction networks from CLIP-seq datasets (22). SomamiR 2.0 contained the cancer somatic mutations in miRNA and competing endogenous RNAs (ceRNAs) including mRNAs, long non-coding RNAs (lncRNAs) and circRNAs (23). PlantCircNet provided visualized plant circRNA-miRNA-mRNA networks specific for plants (24). Most of these databases focused on the identification, expression, evolution or function of circRNAs by high-throughput sequencing technologies. Furthermore, Circ2Traits linked circRNAs and diseases by combining the miRNA-disease associations, disease associated SNPs and Argonaute interaction sites (25). A cancer-specific circRNA database (CSCD) contained the potential cellular localization, miRNA response element sites and RNA binding protein sites, open reading frames and alternative splicing events of parent genes through predicting methods (26). Although Circ2Traits and CSCD have laid a significant foundation for the studies of disease-related circRNAs, these associations are confirmed using computational methods. Therefore, the studies of disease-related circRNAs are still limited and no database focused on the experimentally supported associations between circRNAs and diseases.

To bridge the gap, it is highly desirable to develop a high-quality circRNA-disease association database to study the roles of circRNAs in diseases. Here, we manually curated experimentally validated circRNA-disease associations in circR2Disease from existing literatures prior to 31 March 2018. The final database contains 725 experimentally supported associations between 661 circRNAs and 100 diseases. Furthermore, we summarized the usage of data sets in circR2Disease, which helps to mine the mechanism of the relationship between circRNAs and diseases as well as to predict the novel associations.

## Data collection and database content

The experimentally validated associations between deregulated circRNAs and the occurrence of human diseases were collected through several steps as previously described (27–31). First, we searched the PubMed database with keywords matching ‘circRNA’, ‘circular RNA’, ‘circRNA cancer’, ‘circRNA disease’, ‘circRNA tumor’ and ‘circRNA neoplasm’. Then, we retrieved the entries that describe the associations between circRNAs and diseases manually from these publications. In circR2Disease database, we collected 739 entries that include 725 circRNA-disease associations, 661 circRNAs and 100 diseases from the published papers prior to 31 March 2018. The curated information includes circRNA name, coordinates and gene symbol, disease name, expression patterns of circRNAs (upregulated or downregulated), experimental techniques (qRT-PCR, RNAi, northern blot, western blot, northern blot, luciferase reporter assays and so on), a brief description of circRNAs from literatures, year of publication. At the same time, the hyperlinks to the circBase ID for circRNA, the MalaCards database for disease and the NCBI PubMed ID for the reference are provided. Furthermore, the links of other useful databases are available in the homepage of circR2Disease database, including CircBase, CSCD, TSCD, Circ2Traits and CircInteractome.

Finally, all data in CircR2Disease are stored and managed using SQL Server (version 2008 r2), which is a mid-lighted database management system. The website is developed based on the .Net, a C# web framework (version 4.5). The web service is built using IIS (version 7.0), a Microsoft web service. The CircR2Disease database is freely available at http://bioinfo.snnu.edu.cn/CircR2Disease/.

## User interface

**Figure 1.**
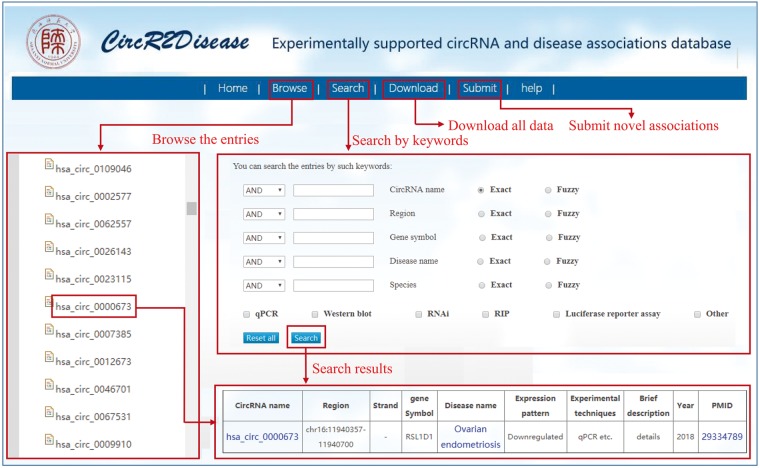
A schematic workflow of CircR2Disease.

The CircR2Disease database provides a user-friendly interface for users to browse, search, download and submit associations between circRNAs and diseases (Figure 1). First, users can browse relevant entries by selecting ‘CircRNA’ or ‘Disease’ on the left to view the corresponding entry. Taking ‘hsa_circ_0005986’ as an example, the page of this entry displays that hsa_circ_0005986 was downregulated in hepatocellular carcinoma. In the search page, users can obtain detailed information on each circRNA-disease associations by inputting the corresponding items such as circRNA name and disease name. CircR2Disease also offers a fuzzy search, whose results will list all potential entries with the full or partial names of keywords. In addition, all data in the database, including circRNA-disease associations, circRNA names and disease names, can be downloaded. The database also provides a submission page, in which users can submit novel identified circRNA-disease associations. Once approved by the review committee, the new associations will be included in the coming release database. Moreover, a detailed tutorial for the usage of the database is available in the ‘Help’ page.

## Discussion and conclusions

Accumulating evidences have revealed that circRNAs are closely correlated with different types of diseases such as atherosclerosis (32), lung adenocarcinoma (33), Alzheimer’s disease (34). Numerous investigations have been carried out to explore the specifically dysregulated circRNAs in diseases, which are considered as promising biomarkers for diagnosis, therapeutic and prognosis. Here, we have developed a database named as CircR2Disease, which integrates the experimentally supported circRNA and disease associations as well as their relationship descriptions. CircR2Disease provides a comprehensive resource for users to browse, search, download and submit the circRNA and disease associations with an easy-to-use web interface.

**Figure 2.**
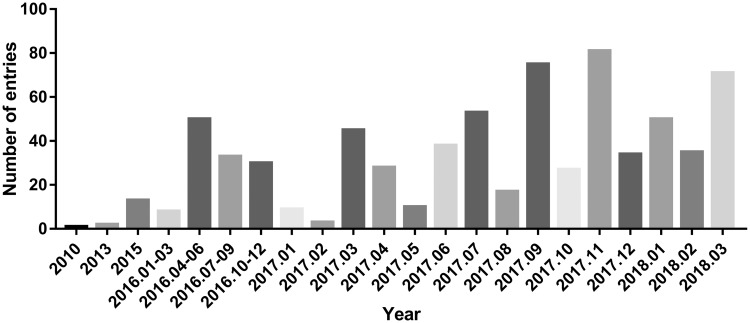
The number of entries in recent years.

**Figure 3.**
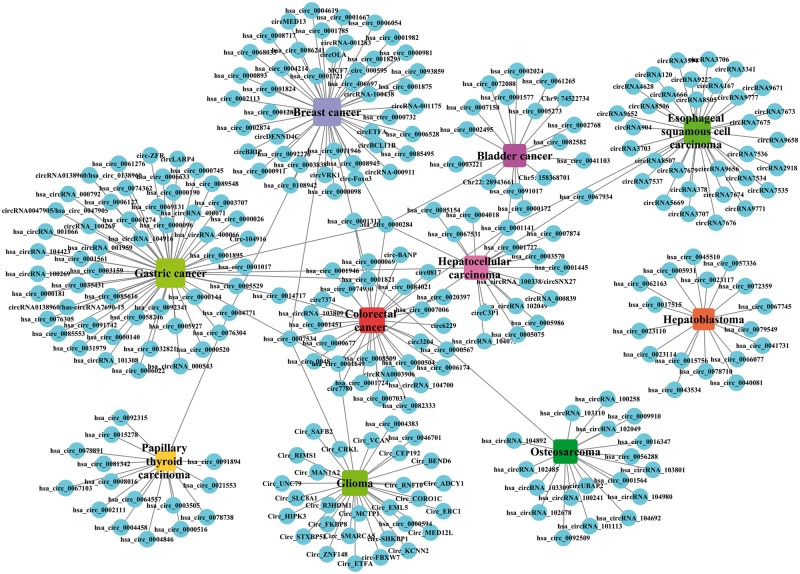
A bipartite network composed of top 10 disease-related circRNAs. Rectangles and circles corresponded to disease names and circRNA names, respectively. An edge corresponds to the experimentally circRNA-disease associations. The size of node corresponds to the degree of these nodes.

**Figure 4.**
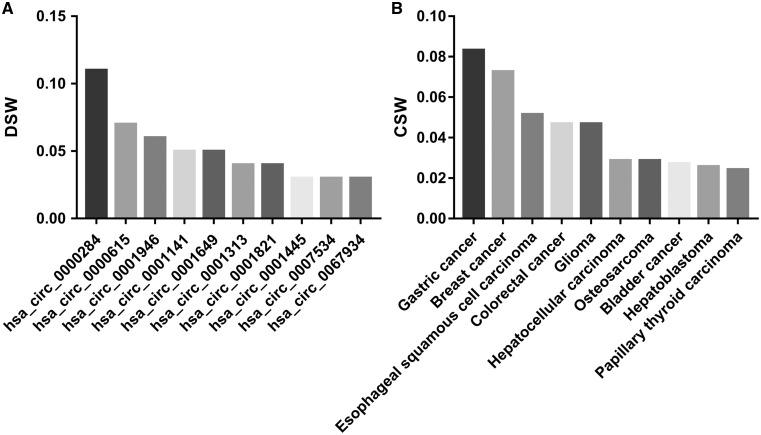
The top 10 circRNAs that with the largest DSWs (**A**) and the top 10 diseases that with the largest CSWs (**B**).

By analysing the entries from CircR2Disease database, we can find the publications about the aberrant expressed circRNA related with diseases are increasing dramatically (Figure 2), which indicates the investigation of the associations between circRNAs and diseases is becoming one of the hot topics. We construct a circRNA-disease bipartite network to describe the top 10 disease-related circRNAs (Figure 3). Based on the network knowledge, we infer that the node with more links was more important, and the loss of this node would have a great influence on the network. With the network, the circRNA hsa_circ_0000284 has the highest connectivity with diseases, and the gastric cancer has the highest connectivity with circRNAs. In addition, the concept of disease spectrum width (DSW) was introduced by a previous study, and we applied it to calculate the DSW of circRNAs (35). For one circRNA *i*, *DSW = n*(*i*)*/N* where, *n(i)* represents the number of disease related with circRNA *i*, *N* represents the total number of diseases related with circRNAs. Here, we used DSW of a circRNA to be a metric to evaluate the importance in diseases. As a result, the top 10 circRNAs with the largest DSWs are shown in Figure 4A. Similarly, the circRNA spectrum width (CSW) of a disease was introduced as a novel metric for one disease and the top 10 diseases with biggest CSWs are listed in Figure 4B. The result shows that the values of DSW and CSW were low, which is mainly because the relationships between circRNAs and diseases are still limited. In addition, the values of DSW and CSW could be acted as two useful metrics with more associations confirmed.

The CircR2Disease database can be widely used to perform specific researches. Based on the hypothesis that circRNAs with similar functions tend to be associated with similar disease phenotypes and vice versa. Researches can predict the potential circRNA-disease associations through bioinformatics methods, such as network-based methods and machine learning methods. In addition, reports have shown that circRNA can perform their biological functions by binding with miRNA (7) and proteins (36). Therefore, miRNA-disease associations and protein–protein interactions are also useful for predicting circRNA–disease relationships. Furthermore, circRNA is a significant class of molecular ceRNAs, the circRNA function may be indirectly inferred by the lncRNA, mRNAs and pseudogenes. Overall, CircR2Disease will be a useful resource for further research of human disease.

## Future extensions

The CircR2Disease provides a high-quality resource for studying the associations between circRNAs and diseases, and further extensions will be developed. It is expected that the number of experimentally validated disease-related circRNAs will continue to increase. The newly validated circRNA-disease associations will be manually curated and CircR2Disease database will be updated every 2 months. Additionally, the experimentally validated circRNA partners including miRNA sponges, proteins or other biological molecules will be integrated. Meanwhile, new tools and algorithms for analysing circRNA-disease associations will be developed and will be integrated into the CircR2Disease database in the future.

## Funding

National Natural Science Foundation of China (grant nos. 61672334, 61502290 and 61401263). Funding for open access charge: National Natural Science Foundation of China (61672334).

*Conflict of interest. None declared.*
